# Conservation genetics of the eastern yellow-bellied racer (*Coluber constrictor flaviventris*) and bullsnake (*Pituophis catenifer sayi*): River valleys are critical features for snakes at northern range limits

**DOI:** 10.1371/journal.pone.0187322

**Published:** 2017-11-02

**Authors:** Christopher M. Somers, Carly F. Graham, Jessica A. Martino, Timothy R. Frasier, Stacey L. Lance, Laura E. Gardiner, Ray G. Poulin

**Affiliations:** 1 University of Regina, Department of Biology, Regina, Saskatchewan, Canada; 2 Saint Mary’s University, Department of Biology, Halifax, Nova Scotia, Canada; 3 University of Georgia, Savannah River Ecology Laboratory, Aiken, South Carolina, United States of America; 4 Royal Saskatchewan Museum, Regina, Saskatchewan, Canada; National Cheng Kung University, TAIWAN

## Abstract

On the North American Great Plains, several snake species reach their northern range limit where they rely on sparsely distributed hibernacula located in major river valleys. Independent colonization histories for the river valleys and barriers to gene flow caused by the lack of suitable habitat between them may have produced genetically differentiated snake populations. To test this hypothesis, we used 10 microsatellite loci to examine the population structure of two species of conservation concern in Canada: the eastern yellow-bellied racer (*Coluber constrictor flaviventris*) and bullsnake (*Pituophis catenifer sayi*) in 3 major river valleys in southern Saskatchewan. Fixation indices (F_ST_) showed that populations in river valleys were significantly differentiated for both species (racers, F_ST_ = 0.096, P = 0.001; bullsnakes F_ST_ = 0.045–0.157, P = 0.001). Bayesian assignment (STRUCTURE) and ordination (DAPC) strongly supported genetically differentiated groups in the geographically distinct river valleys. Finer-scale subdivision of populations within river valleys was not apparent based on our data, but is a topic that should be investigated further. Our findings highlight the importance of major river valleys for snakes at the northern extent of their ranges, and raise the possibility that populations in each river valley may warrant separate management strategies.

## Introduction

The genetic population structure of snakes can vary markedly based on a number of intrinsic and extrinsic factors [1–3]. Some snake species show only very modest levels of subdivision or none at all (e.g. *Rhinoplocephalus nigrescens*; [4], *Crotalus horridus*, [5]), while others show a high degree of differentiation over small spatial scales (e.g. *Sistrurus catenatus catenatus*; [6], *Nerodia erythrogaster neglecta*; [7], *Vipera berus*, [8]; *Coronella austriaca*, [9]; *Crotalus triseriatus*, [10]). The variation among species is likely due to a wide variety of biological traits (e.g. natal philopatry, home range size, specific habitat requirements), as well as the ability of individuals to disperse. Dispersal and associated gene flow among breeding groups may also be influenced by extrinsic factors such as natural or man-made barriers [11]. For example, Marshall et al. [7] showed that anthropogenic habitat loss caused enhanced genetic isolation in the copperbelly water snake (*Nerodia erythrogaster neglecta*, a habitat specialist) by hindering dispersal. It is clear that intrinsic and extrinsic factors may interact to produce constraints to dispersal and gene flow in snakes; however, most studies have addressed populations at the core of known geographic ranges (e.g. [12]). It is only recently that snake populations at range peripheries (e.g., [13–15]) or in extreme environments (e.g., [16]) have become the focus of conservation genetics studies. Conservation challenges may be exacerbated for populations at range peripheries, where extreme environments and naturally sparse distributions interact with anthropogenic activities to generate additional risk factors.

Snake populations at northern range limits face ecological challenges that may affect dispersal, gene flow, and ultimately genetic population structure. For example, snakes at higher latitudes in North America and Eurasia rely heavily on a limited number of suitable hibernacula to survive harsh winters [17], and they often exhibit high fidelity to these sites (e.g. *Elaphe obsoleta obsoleta*; [18], *Gloydius halys*; [19]). In addition, landscapes containing both suitable hibernacula and summer habitat for northern snakes (e.g., [20]) may be patchily distributed at range margins, a situation that is exacerbated by human activities that cause habitat loss and fragmentation [21]. Thus, gene flow may only occur among northern snake populations when individuals travel long distances away from dens to breed; however, successful dispersal may be uncommon, resulting in highly subdivided populations. Interestingly, several recent studies have shown that some snake species have much larger home ranges and travel long distances from hibernacula at northern range limits (e.g. [20, 22–24]). In principal, these behavioural traits may partially counteract the barriers to gene flow discussed above. Understanding this situation is of key interest in Canada, where a variety of North American snakes, some of which are of conservation concern, reach their northern range limits [25].

The Great Plains in central Canada represents the northern range limit for the eastern yellow-bellied racer (*Coluber constrictor flaviventris*, hereafter racer) and the bullsnake (*Pituophis catenifer sayi*). In addition to being the range periphery for these species, the northern Great Plains is also one of the most human-altered landscapes in North America; over 70% of native grassland has been lost due to agriculture, and the region contains a high density of species at risk of extinction or extirpation [26]. Racers and bullsnakes have distributions in Canada that are linked to major river valleys in southern Saskatchewan and Alberta. Almost nothing is known about population size, structure, or the degree of isolation of either species in this region. Both racers and bullsnakes have specific habitat requirements, including hibernacula with particular characteristics (thermal properties), and they often exhibit a high degree of fidelity to these sites [20,23]. Large geographic distances separate river valleys and hibernacula, which may be further isolated due to extensive land conversion for agriculture, creating an inhospitable landscape for snake dispersal. Both species are considered vulnerable to extirpation in Canada due to limited habitat availability and conflict with humans. The racer is currently listed as a Threatened species [27], and the bullsnake is considered of special concern due to a nearly complete lack of basic knowledge about their populations, and documented conservation threats [28]. The connectivity among occupied sites in Canada, and the potential for genetic differentiation among locations has not been previously investigated for these species, but is critical knowledge to facilitate on-going conservation planning.

Here we use microsatellite loci to examine the genetic population structure of racers and bullsnakes in Saskatchewan, Canada. Specifically, we test the hypothesis that snake populations in the major river valleys are genetically distinct groups. Snakes likely colonized the northern Great Plains via northward dispersal along river valleys from more contiguous parts of their range in the U.S.A. The population in each valley may thereby have originated from independent founder events. In addition, these founder groups have likely been isolated from one another for extended periods of time due to a lack of suitable habitat between river valleys and the restricted movement and dispersal ability of the snake species [23]. Thus, we predicted that populations in each river valley would be well differentiated from other such groups. Ultimately, our goal was to identify the appropriate scale for defining management units for racers and bullsnakes. Our work represents the first genetic study of these snake species on the northern Great Plains.

## Methods

### Study area and sample collection

**Fig 1:**
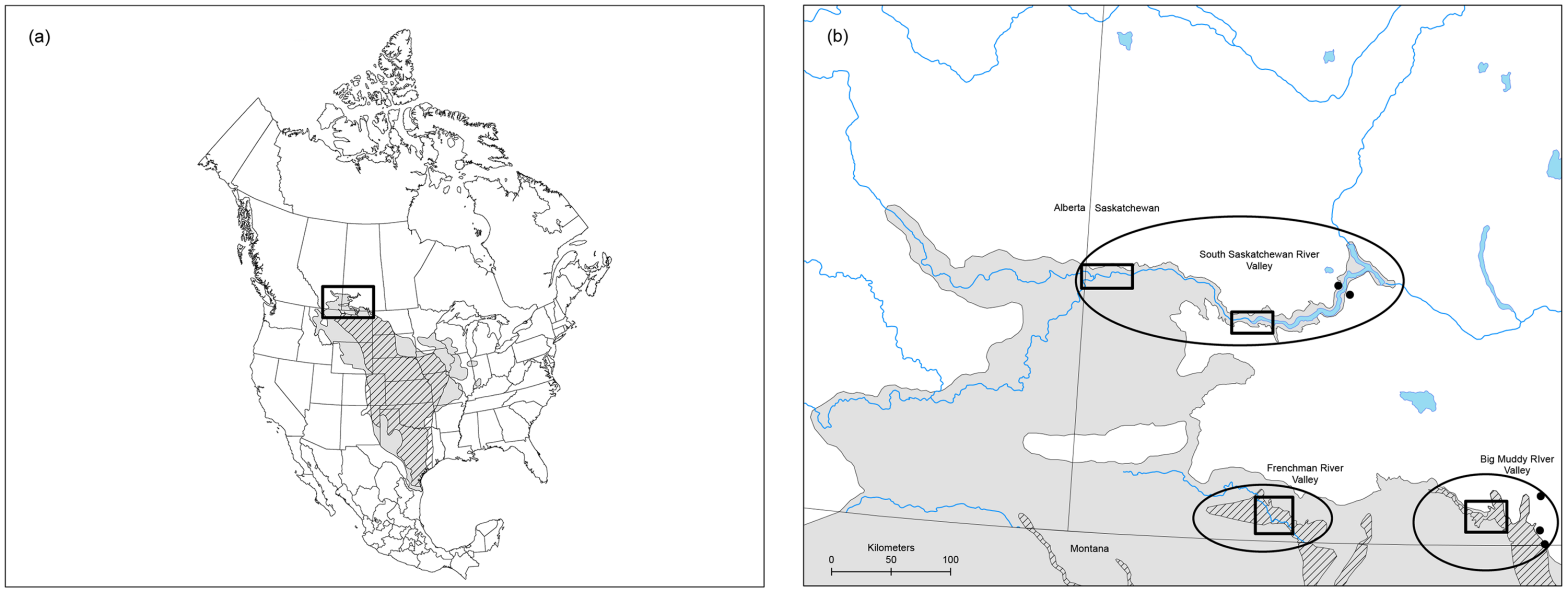
(a) The range of eastern yellow-bellied racers (*Coluber constrictor flaviventris*) and bullsnakes (*Pituophis catenifer sayi*) in North America. Light grey represents the range of bullsnakes, dark grey represents the range of racers, and diagonal lines indicate range overlap. The square outlines our study area in Saskatchewan, Canada. (b) Expanded view of the study area highlighting the 3 major river valleys (Frenchman River Valley [FRV], South Saskatchewan River Valley [SSRV], and Big Muddy River Valley [BMRV]). The specific study areas encompassing sample locations within the valleys are highlighted as boxes; the locations of isolated snakes used in analysis are presented as dots.

Racers and bullsnakes have broad distributions in North America, but are only found in a very small portion of southern Canada where they reach their northern range limits (Fig 1a). Our study sites were located in southwestern Saskatchewan, where these species appear to be concentrated in three large river valleys: (1) the Frenchman River Valley (FRV; both species), (2) the Big Muddy River Valley (BMRV; both species), and (3) the South Saskatchewan River Valley (SSRV; bullsnakes only; Fig 1b). These river valleys represent almost the complete known Canadian range of racers [29], and a large portion of the Canadian range for bullsnakes [25]. Our study species are sympatric in the grasslands of Saskatchewan, hibernating communally in dens located in bluffs of river valleys. Hibernacula appear to persist for long periods of time (decades), but are vulnerable to erosion and other factors that cause them to become suddenly unavailable to snakes [30]. Connectivity between river valleys has not been previously investigated, but is critical to understand for conservation planning. Agricultural habitats are avoided by both species [20,23], which may exacerbate long-standing isolating factors. Individuals occupying each river valley may therefore represent genetically differentiated populations that require individual management strategies. Destruction of even one den and its associated snakes (as in [30]), or extirpation of snakes from the few occupied dens in a single river valley, may represent major losses to the Canadian population with little chance of recovery due to rescue dispersal.

For genetic analyses, we collected blood samples from live snakes caught by hand during foot surveys or in drift fences with traps during spring emergence from hibernacula in 2007–2013 (described in [20,23]). We captured most live snakes after emergence from 18 known hibernacula in the 3 river valleys: 9 sites in the FMRV, 5 sites in the BMRV, and 4 sites in the SSRV (see S1 Table for site locations). However, some blood samples were collected opportunistically from snakes encountered away from den sites during other activities. For this study we were interested primarily in population structure at the river valley level. From captured snakes, we drew blood (25–100 μl) from the caudal vein using a 27-gauge needle, or clipped a small portion of tissue from the distal end of the tail (< 5mm) using a sterile razor blade. We also collected tissue from road-killed snakes in the study areas; from these snakes we excised a small portion of dorsal muscle tissue. Blood and other tissue samples were stored in lysis buffer (4.0 M urea / 0.2 M NaCl / 0.1 M Tris–HCl, pH 8.0 / 0.5% n-laurylsarcosine / 0.1 M 1,2-cyclohexanediamine) at 4°C until DNA extraction. All animal procedures were approved by the President’s Committee on Animal Care at the University of Regina, according to guidelines determined by the Canadian Council on Animal Care. Permits were provided by the Saskatchewan Ministry of the Environment (Scientific Sampling Permit), and Environment Canada (Species at Risk Permit).

### Genetic analyses

We extracted DNA using DNeasy spin-column kits according to the manufacturer’s guidelines (Qiagen Inc., Ontario, Canada); however, proteinase K digestions were extended to 8–12 hours at 56°C and we performed the optional RNase A treatment (Qiagen Inc., Ontario, Canada). DNA was quantified using a spectrophotometer (NanoDrop 1000, Thermo Scientific, Wilmington, DE, USA). We genotyped all individuals (n = 177 racers, n = 103 bullsnakes) at 10 microsatellite loci that had been previously developed for each species (racers, [31]; bullsnakes, [32]). PCR was performed in 25 μl reactions containing 1X PCR Master Mix (Norgen Biotek, Mississauga, ON, Canada), 2 μM forward and reverse primer (forward primer labelled with fluorescent marker) and 10 ng of template DNA. For racers, the thermal cycling was conducted as follows: 94°C for 5 min; 30 cycles of 94°C for 30 s, annealing temperature for 45 s, 72°C for 45 s; 8 cycles of 94°C for 30 s, 53°C for 45 s, 72°C for 45 s; and a final extension step of 30 min at 72°C. For bullsnakes, either standard thermal cycling parameters or a touchdown protocol was used depending on the locus (see [32]). Standard thermal cycling parameters were conducted as follows: 94°C for 5 min, followed by 40 cycles of 96°C for 30 s, annealing temperature for 30 s, and 72°C for 30 s. Touchdown cycling parameters consisted of 95°C for 5 min, 20 cycles of 96°C for 30 s, annealing temperature of 65°C (decreasing 0.5°C per cycle to 55°C) for 30 s, 72°C for 30 s, and 20 cycles of 96°C for 30 s, 55°C for 30 s, and 72°C for 30 s. Both positive and negative controls were run for all loci; template DNA from a well-characterized individual was used as a positive control.

PCR products were size-fractionated using capillary electrophoresis on a DNA sequencer (Beckman-Coulter GeXP). A 600-bp in-lane size standard was used to determine the size of fragments with single base pair resolution (Beckman-Coulter, Fullerton, CA, USA). We scored microsatellite alleles with the aid of GENEMARKER v2.2.0 software (SoftGenetics, State College, PA, USA) using default settings, with the exception that our bin width was expanded to ±1 base pair to reflect the resolution limits of the sequencer. All microsatellite profiles were visually inspected to confirm the accuracy of calls; a second observer independently verified the scoring, and both observers were blind to the geographic origin of samples. We quality checked microsatellite data sets and estimated the potential frequency of null alleles using MICRO-CHECKER [33]. We used GenAlEx [34] to estimate the observed and expected heterozygosities (H_O_ and H_E_), number of alleles per locus, and total number of private alleles. We examined deviations from Hardy-Weinberg Equilibrium (HWE) using GENEPOP v4.1.4 [35]. We estimated the inbreeding coefficient (F_IS_; [36,37]) using FSTAT [38]. The complete microsatellite dataset has been deposited on Dryad (http://dx.doi.org/10.5061/dryad.cc6r3).

We used several approaches to assess population structure in our data sets. In the first, we designated populations based on river valleys and compared fixation indices (F_ST_) using Analysis of Molecular Variance (AMOVA; [36,37]) in the program GENODIVE [39]. Second, we used Bayesian clustering in the program STRUCTURE [40,41] with an admixture model and correlated allele frequencies to perform unsupervised clustering of the whole data set for each species. We repeated analyses 10 times for each value of K ranging from 1 to 10, and employed a burn-in time of 100,000 with 1,000,000 MCMC steps. We calculated ΔK using the methods of Evanno et al. [42] implemented in STRUCTURE HARVESTER [43] to determine the most likely number of clusters. When subdivision was identified, we analyzed the groups hierarchically using STRUCTURE to examine finer-scale subdivision. Similar to global analyses, the optimal number of groups was inferred using ΔK. We used the program CLUMPP to determine the optimal assignments of individuals to clusters [44]. Graphical displays of STRUCTURE findings were generated using the DISTRUCT program [45]. Lastly, we analyzed the data sets using Discriminant Analysis of Principal Components (DAPC), a multivariate ordination approach from the R package *adegenet* [46,47]. DAPC does not require the assumption of HWE and uses ordination to maximize the between group variation while minimizing the variation found within groups. DAPC requires imputation to eliminate missing data. Both datasets had missing data with bullsnakes missing 4.8% of genotypes and 3.0% missing in racers. We imputed missing genotypes for DAPC using a random forest approach with 100 trees and 100 iterations using the *stackr* program in R [47,48]. Ellipses were generated using the optimal number of principal components as determined by the function *dapc_a_score*, which was 13 and 17 for bullsnakes and racers, respectively.

## Results

Assessment in MICROCHECKER did not indicate issues with large allele drop-out or stutter, but identified a high probability of null alleles in both species. Two loci likely had a high frequency of null alleles for racers (CCPKZ06 and CCPKX22), and one locus in bullsnakes (Piru15); these loci were removed from further analyses. Tests for HWE on the overall data set revealed that there was a significant excess of homozygotes at 7 of 9 loci for bullsnakes and 4 of 8 for racers after sequential Bonferroni correction. However, given the consistent and pervasive nature of the homozygote excess across loci and species, and the small number of individuals that failed to PCR amplify (potential homozygous nulls), we concluded that the deviation from HWE was most likely due to population structure in the data set (Wahlund effect). Thus, 9 and 8 loci were retained for subsequent analyses of population subdivision for bullsnakes and racers, respectively.

### Racers

**Fig 2:**
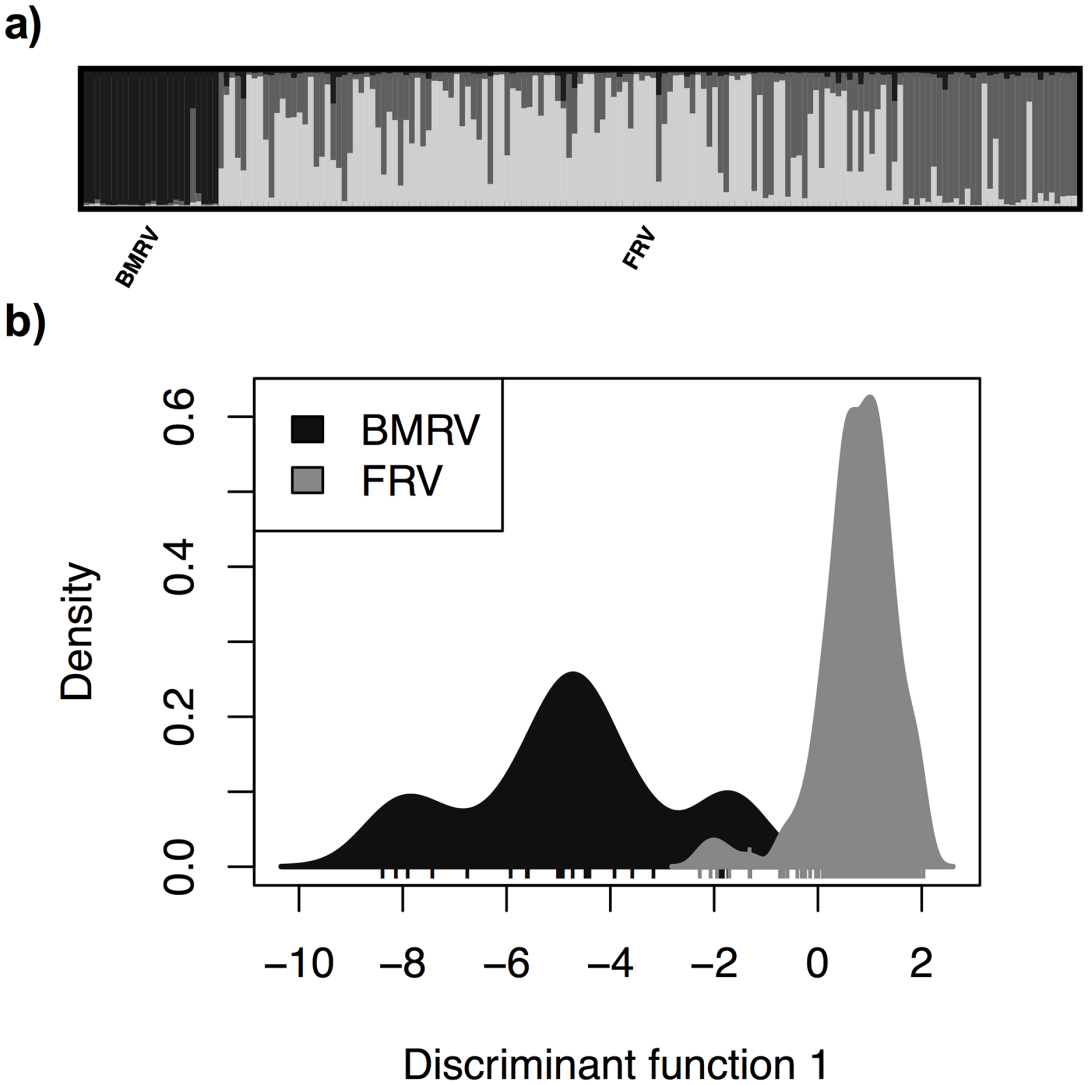
(a) The *distruct* plot of K = 3 from STRUCTURE analysis of eastern yellow-bellied racers in Saskatchewan, Canada. Each river valley was also run separately in STRUCTURE and did not show any distinct clusters. (b) DAPC analysis showing the first two discriminant functions. The DAPC analysis included the 5 den sites from the Frenchman River Valley with large enough sample sizes (min. of 5 snakes) to be considered individually.

Fixation index analysis based on user-defined populations showed significant differentiation between the FRV and BMRV (F_st_ = 0.08, P = 0.001). Both areas also had high numbers of private alleles (Table 1). Results from STRUCTURE confirmed the finding of multiple populations, identifying K = 3 as having the highest probability (using the ΔK approach). Visualization of groupings in DISTRUCT clearly separates the FRV and the BMRV from one another with Q>0.94 for samples in the BMRV (Fig 2a). The K = 3 value is generated by an apparent second cluster within the FRV, with Q values of 0.56 and 0.42 for each of the clusters; however, this potential substructure within the FRV is not explained by den site or geography (data not shown). To further disentangle this substructure, we ran the FRV separately in STRUCTURE, which yielded a most likely number of clusters of K = 1. Thus, any substructure within the FRV may be very weak, and not resolvable with our current data. The DAPC analysis further confirmed clear differentiation by river valley across the first discriminant function (Fig 2b). F_IS_ values were similar (0.037 and 0.055) for both the BMRV and FRV overall.

**Table 1:** Number of individuals sampled (N), average number of alleles per locus observed (A_N_), number of private alleles (A_P_), and observed and expected heterozygosity (H_O_ and H_E_) for eastern yellow-bellied racers and bullsnakes in Saskatchewan, Canada. Data presented are for each population at 8 microsatellite loci for racers and 9 loci for bullsnakes.

Species	Population	N	A_N_	A_P_	H_O_	H_E_
Racer	FRV	153	12.9	60	0.73	0.77
	BMRV	24	7.5	17	0.76	0.77
Bullsnake	FRV	48	13.2	36	0.81	0.87
	BMRV	21	9.2	15	0.78	0.81
	SSRV	34	7.8	19	0.53	0.69

### Bullsnakes

**Fig 3:**
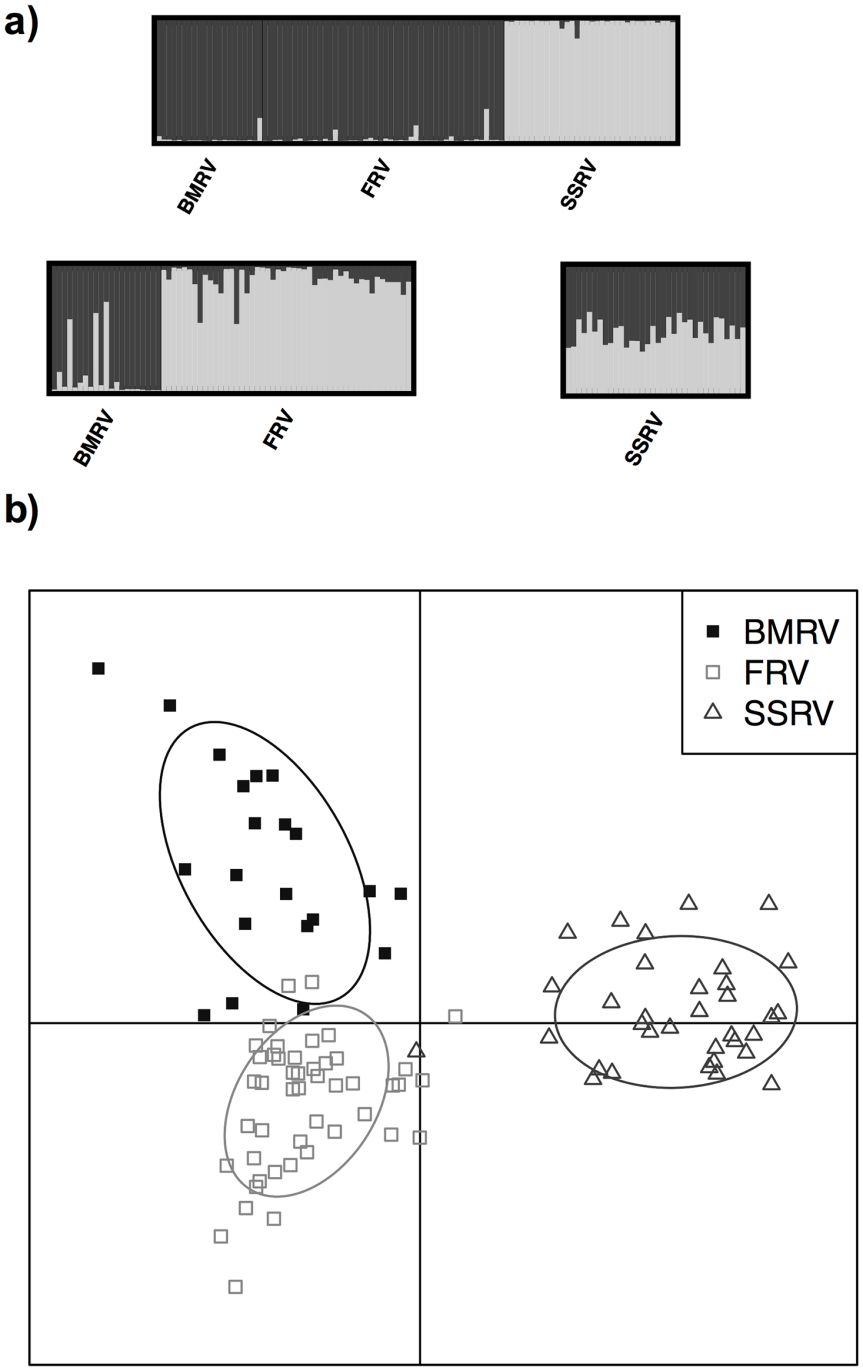
(a) The *distruct* plots generated from STRUCTURE analysis and (b) DAPC analysis of bullsnakes in Saskatchewan, Canada. STRUCTURE was run hierarchically and additional structure was found when the SSRV data were removed (K = 2 between FRV and BMRV). No further structure was detected within each of the river valleys. The DAPC analysis shows the first two discriminant functions between the river valleys.

F_st_ analysis based on user-defined populations showed significant differentiation between all three river valleys (Table 2). The highest levels of differentiation were between the population in the SSRV and those in the other two areas (F_st_ values as much as 3x higher). All three bullsnake populations also had a large number of private alleles, which further reinforces the distinction among river valleys (Table 1). Population subdivision by river valley was confirmed by Bayesian clustering using STRUCTURE, which identified the optimal value of K = 2 in the global analysis (based on ΔK), with the SSRV forming a distinct cluster from the BMRV and FRV (Q>0.98; Fig 3a). STRUCTURE was run with the data from the SSRV removed, and the optimal value was K = 2, differentiating the FRV and BMRV groups (Q>0.87; Fig 3a). When both the SSRV and BMRV were run individually the optimal value was K = 1 for both areas. Differentiation among the three river valleys was further confirmed using DAPC (Fig 3b). The first discriminant function resolved the population in the SSRV from those in the FRV and BMRV; the second discriminant function resolved populations in the BMRV and FRV from each other. The observed F_IS_ values were 0.074, 0.241, and 0.073 for the FRV, SSRV and BMRV, respectively, indicating substantially higher levels of inbreeding in the SSRV.

**Table 2:** F_ST_ values for pair-wise comparisons of bullsnake populations in the Big Muddy River Valley (BMRV), the Frenchman River Valley (FRV), and the South Saskatchewan River Valley (SSRV) in Saskatchewan, Canada. The F_ST_ values are found below the diagonal and the corresponding p-values are found above the diagonal.

	BMRV	FRV	SSRV
BMRV	-	0.001	0.001
FRV	0.045	-	0.001
SSRV	0.156	0.136	-

## Discussion

We examined the population structure of racers and bullsnakes at their northern range limits and found that river valleys contained genetically differentiated populations of each species. The differentiation we observed could be the result of independent colonization of the valleys via northward movements from the range core, or the result of population discontinuity generated by historical habitat loss between valleys. Our data cannot discriminate between these two scenarios, but in either case the restricted ability of snakes to disperse out of the river valleys would limit gene flow, resulting in isolation and population differentiation. There are large geographic distances separating occupied sites within each of the three river valleys, and it is unlikely that there is currently appropriate habitat (e.g., hibernacula) between sites to allow for population continuity. In addition, dispersal may be further restricted by human activities; both racers and bullsnakes avoid agricultural land [20,23], which is prominent in the areas between river valleys. Some snake species can maintain gene flow even across large geographic distances (e.g. *Natrix natrix*; [21]), but in these situations, corridors of suitable habitat are present to facilitate the movement of individuals between isolated patches. The isolation of racer and bullsnake populations in separate river valleys on the northern Great Plains suggests that there is little possibility of natural rescue dispersal from existing populations within Canada in the event of local extirpations. However, the level of connectivity to larger contiguous populations in the USA is unknown and requires further research. Similar to the situation with other northern snake populations (e.g., [14,15]), our findings suggest that population subdivision on a regional scale is an important consideration for conservation planning.

Our analyses suggest that both racers and bullsnakes exhibit no, or only very weak, fine-scale structure within the FRV based on overwintering hibernacula (dens). Fine-scale population structure over short geographic distances is common in snakes, particularly species that are habitat specialists or exhibit philopatry to communal dens (e.g. [5,8,18]; reviewed by [1]). Bayesian clustering identified two potential clusters for racers in the FRV, but these groupings were not based on den site, and the clusters were not supported by DAPC analysis. We performed one comparison of bullsnakes from two dens in the BMRV, and they were not differentiated (data not shown). Thus, at this point we conclude that it is likely river valleys, rather than individual hibernacula, that are the important unit of population subdivision for our study species. However, we suggest that future studies attempt to collect additional samples from more dens to provide more data on fine-scale patterns. This may be particularly important for bullsnakes in the SSRV, which have much higher F_IS_ values than those from the FRV and BMRV, suggesting that inbreeding may lead to more fine-scale structure. In addition, other genetic markers (e.g., single nucleotide polymorphisms) might also enhance ability to identify fine-scale structure.

Racers and bullsnakes in Canadian river valleys are different from those that are part of more contiguous populations in the USA, and may therefore have additional conservation value. Racers in Saskatchewan are less genetically diverse than those at the core of their range. For example, Klug et al. [12] observed high allelic diversity and high heterozygosity (mean number of alleles/locus = 20.9, mean H_E_ = 0.83) at the same microsatellite loci in Kansas racers, while Canadian racers in our study had much lower allelic diversity and heterozygosity (mean number of alleles/locus = 7.5–12.9, mean H_E_ = 0.77). Thus, populations at the northern range periphery are less genetically diverse than at the range core. Local biotic and abiotic conditions can drive adaptation in snakes (e.g. [16,49,50]), and although the microsatellites we used are neutral makers, we propose that genetic drift and selection have created unique populations of snakes capable of occupying the extreme environments they face in southern Canada (local adaptation). The notion of local adaption is also supported by ecological and behavioural differences; racers at the core of their range are habitat generalists and do not exhibit high levels of fidelity to dens [12]. In contrast, racers at northern range limits in Canada are habitat specialists and exhibit high levels of fidelity to hibernacula, at least over the short term. In addition, racers in Canada also have very large home ranges and move farther from dens to reach summer habitats than other more southerly populations [20,23]. Bullsnakes have been poorly studied so there are few data available for comparison; however, due to their similarity to racers in our study, it is likely that Canadian bullsnake populations are also different from those at the core of their range. Little is known about the general conservation significance of peripheral populations of snakes, but due to their potential uniqueness and susceptibility to extirpations, more studies are clearly necessary.

### Implications for conservation

The eastern yellow-bellied racer has a federal status of Threatened in Canada and is therefore the subject of active conservation planning [27,51]. It is important to know whether this species needs to be managed as a single entity or multiple designatable units (DUs). Other recent studies have revealed critical mismatches between the number and extent of snake populations based on genetic markers, and the scale of conservation plans or management units (e.g., [15,52]). According to guidelines from the Committee on the Status of Endangered Wildlife in Canada (COSEWIC), the advisory body to the federal Minister of the Environment, populations warrant separate DU status if they are both ‘discrete’ and ‘significant’ [53,54]. Based on our microsatellite data and the limited opportunity for dispersal, racer populations in major river valleys in Saskatchewan are certainly differentiated, but their significance is less clear. We suggest that racers have local adaptations to their extreme northern environments, potentially satisfying COSEWIC DU criterion #2: ecological setting likely or known to have given rise to local adaptations. However, it is important to recognize that the comparison we have drawn here is to core populations much farther south in the USA; it is less likely that populations have unique local adaptations among individual river valleys within Canada. Given that racers have confirmed populations in only a few river valleys in all of Canada, the loss of any one of these groups would satisfy COSEWIC DU criterion #4: loss of discrete population results in an extensive gap in the range of the species in Canada. However, recent confirmation of racers in the east block of Grasslands National Park between the FRV and BMRV (R.G. Poulin, unpublished data), and a confirmed hibernaculum in extreme south-eastern Alberta hint at the possibility of a broader Canadian distribution (see [29]). Thus, at this time we cannot make a firm recommendation about racer DUs.

Bullsnakes have a status of Special Concern in Canada because of documented conservation threats and a nearly complete lack of information regarding the population size of this species. Bullsnakes may be vulnerable to many of the same factors that affect racers, which are sympatric for a large portion of their Canadian range [23]. Our genetic data show similar patterns of population structure among groups of bullsnakes in major river valleys, again indicating that they are differentiated, and that movement and gene flow between river valleys is limited. The status of Special Concern does not currently carry legal protection for the bullsnake, so there is little point in considering the potential validity of Canadian DUs for this species. However, as more information becomes available the status assessment for bullsnakes should consider features of their genetic population structure. The SSRV may be particularly important given that it spans the provinces of both Saskatchewan and Alberta, and hosts a population of bullsnakes that is highly differentiated from others in Canada.

Despite uncertainty about the significance of differentiated racer and bullsnake populations, it is important to consider that populations of both species are vulnerable to stochastic events at hibernacula (see [30]), and also to human changes to grassland habitats. The potential influence of humans on these two snake species varies markedly by location, and may change further over time. For example, at the time of this study, the majority of land in the FRV occupied by racers and bullsnakes was protected inside of Grasslands National Park (49,000 Ha) and a federal community pasture belonging to Agriculture and Agri-Food Canada (41,000 Ha). However, the community pasture program was recently discontinued, potentially jeopardizing the protected status of 46% of the range of the racer in the FRV. The fate of the community pasture land, despite its importance to Canadian species at risk of extinction, is uncertain. In contrast to the FRV, all land in the BMRV is privately owned or leased, offering no formal protection of important habitat for snakes or other wildlife species. Fortunately, most land in the BMRV is currently used for livestock ranching, which has much less impact on grasslands than conversion to cereal crops. The SSRV is a much larger geographic feature, and is currently a mosaic of land uses with only very small areas of formally protected habitat (e.g., Saskatchewan Landing Provincial Park). Major differences in the amount of protected land suggest that long-term conservation planning may have to be tailored to each of the river valleys. This need may tip the balance in favour of formal DUs for racers despite some ambiguity as indicated above.

## Supporting information

S1 Table Locations and snake species present for sites sampled in the Frenchman, Big Muddy, and South Saskatchewan River valleys for this study. To protect sensitive habitat and over-wintering hibernations sites for these species of conservation concern in Canada, exact locations have been offset by several hundred meters. Eastern yellow bellied racer = *C*.*c*. *flaviventris*; bullsnake = *P*.*c*. *sayi*.
(DOCX)
