# Supplementary material for: Conservation genetics of the eastern yellow-bellied racer (Coluber constrictor flaviventris) and bullsnake (Pituophis catenifer sayi): River valleys are critical features for snakes at northern range limits
Source: PLoS One. 2017 Nov 2;12(11):e0187322. doi: 10.1371/journal.pone.0187322 (PMC5667752; doi:10.1371/journal.pone.0187322)
Supplement: S1 Table — To protect sensitive habitat and over-wintering hibernations sites for these species of conservation concern in Canada, exact locations have been offset by several hundred meters. Eastern yellow bellied racer = C.c. flaviventris; bullsnake = P.c. sayi. (DOCX) [file pone.0187322.s001.docx]

Table S1. Locations and snake species present for sites sampled in the Frenchman, Big Muddy, and South Saskatchewan River valleys for this study. To protect sensitive habitat and over-wintering hibernations sites for these species of conservation concern in Canada, exact locations have been offset by several hundred meters. Eastern yellow bellied racer = *C.c. flaviventris*; bullsnake = *P.c. sayi*.

| River Valley | Site | Latitude | Longitude | Species Sampled |
| --- | --- | --- | --- | --- |
| Frenchman | FRV1 | 49.181 | -107.572 | *C.c. flaviventris* |
|  | FRV2 | 49.133 | -107.331 | Both |
|  | FRV3 | 49.218 | -107.704 | Both |
|  | FRV4 | 49.316 | -107.811 | Both |
|  | FRV5 | 49.351 | -107.806 | Both |
|  | FRV6 | 49.208 | -107.713 | Both |
|  | FRV7 | 49.241 | -107.714 | Both |
|  | FRV8 | 49.146 | -107.820 | *C.c. flaviventris* |
|  | FRV9 | 49.452 | -107.515 | *C.c. flaviventris* |
| Big Muddy | BMRV1 | 49.213 | -105.228 | Both |
|  | BMRV2 | 49.188 | -105.161 | Both |
|  | BMRV3 | 49.389 | -105.635 | *P.c. sayi* |
|  | BMRV4 | 49.456 | -104.607 | *C.c. flaviventris* |
|  | BMRV5 | 49.164 | -104.586 | *C.c. flaviventris* |
| South Saskatchewan | SSRV1 | 50.883 | -109.544 | *P.c. sayi* |
|  | SSRV2 | 50.917 | -106.8890 | *P.c. sayi* |
|  | SSRV3 | 50.648 | -107.962 | *P.c. sayi* |
|  | SSRV4 | 50.656 | -107.983 | *P.c. sayi* |
